# Transport equipment network analysis: the value-added contribution

**DOI:** 10.1186/s40008-022-00289-1

**Published:** 2022-12-06

**Authors:** Luis Gerardo Hernández García

**Affiliations:** Graduate School of Economics, Kyushu University, Fukuoka, Japan

**Keywords:** Network analysis, Value-added, Community detection, Centrality measures, Transport equipment, C38, C45, F1, F14

## Abstract

Emerging in the twenty-first century, Network Science provides practical measures to interpret a system’s interactions between the components and their links. Literature has focused on countries’ interconnections on the final goods, but its application on the value-added from a network perspective in trade is still imitated. This paper applies network science properties and a multi-regional input–output analysis by using the UNCTAD-Eora Global Value Chain Database on the Transport Equipment value added on 2017 to unwrap the specific structural characteristics of the industry. Results show that the industry is highly centralized. The center of the network is dominated by developed countries, mainly from Europe, the United States, and Japan. Emerging countries such as China, Mexico, Thailand, and Poland also have an important position. In addition, the structure reveals two sub-hubs located in East Europe and North America. By extending to community detection, the network consists of three different communities led by Germany, the United States, and the United Kingdom, associated with more significant value-added flows. The study concludes that flows are not always consistent with the economy’s geographical location as usually final goods analysis suggests, and highlight the need to continue using the complex network to reveal the world trade structure.

## Introduction

The increase in technological processes and reduction in the production and communication cost have transformed the international trade structure into a more dynamic and fragmented system. Thus, it has created different opportunities for diverse economies to be immersed in the Global Value Chains (GVCs). One of these opportunities is present in the manufacturing sector, specifically in the transport equipment industry, due to the rapid acceleration in the automobile sector, their capacity for re-allocation and the Foreign Direct Investment (FDI) flows.

Due to this transformation, a simple analysis of final imports and exports is no longer sufficient to evaluate an economy’s strengths and weaknesses. This type of analysis limits itself by not being able to detect how many economies were related by transforming the final product and due to the challenges of collecting data about the value-added for different products, very few organizations and studies have addressed this phenomenon.

Fortunately, network analysis is an efficient tool to represent and examine different systems and their interdependency. The approach has been used widely in various fields, including neuroscience (Strogatz 2001), biology (Buchanan et al. 2010; Leyeghifard et al. 2017), environment (Kagawa et al. 2015; Chen et al. 2018; Chai et al. 2011), and to less extent, in economics (Brailly 2016; Kitsak et al. 2010).

Barabási (2016) defines a network as a “catalog of a system’s components often called nodes or vertices and the direct interactions between them call links or edges.” As Amador and Cabral (2016) suggest, networks suppose the interdependence of observable [and non-observable] data and inspect the influence of connections instead of being treated as isolated agents. In addition, the importance of network analysis relies on the study of relations in a structural form; that is, it considers the effects of a third party on the relationship of *i* and *j*, and how this can influence others.

In the context of trade, Synder and Kick (1979) are one of the early studies using network analysis. They examine the world system theory in line of the center and periphery during the 1980s; however, the conceptualization of the rise of globalization cannot be explained. In a recent study, Kali et al. (2013) considered a country’s specialization patterns to unwrap the trade network system, finding that density and proximity are relevant variables for a country to move to higher-income products; therefore, higher growth rates.

Noguera et al. (2016) applied a traditional input–output (IO) and network analysis, concluding that many economic sectors are related because they present similar characteristics in all countries. They concluded that development level implies an increasing concentration of economic activity in more and better-connected sectors.

Gala et al. (2018) pointed out that countries at the core tend to specialize in producing and exporting goods with high value-added (complex goods), being those countries in their majority high-income countries, while the periphery consists in low-income countries and with low technology and complexity products. More recently, Gould et al. (2018) found that the main channels to determine growth in the trade networks are the FDI flows, migration, and the internet from a multidimensional connectivity perspective. Zhou (2020), analyzing the Regional Trade Agreement (RTA) effects, suggested that two trading partner countries located in the center of the network and with an RTA have a more significant trade level than those in the periphery. Vidya and Prabheesh (2020), in the situation of the COVID-19 pandemic, concluded that emerging Asian economies, such as China and India, have taken the lead roles in the world trade networks.

Therefore, network analysis can be applied in the GVCs context to examine the structure, connectivity and dependence, the countries participating in, and their dominance, a different perspective from the world system theory, which focuses only on identifying the core and periphery without analyzing the interdependency among members.

In that sense, Amador and Cabral (2016), using the World Input–Output Database, analyzed the evolution of the degree centrality in a weighted directed network. They concluded that more countries are joining the GVC as the density of the different networks increases within years. Moreover, Cerina et al. (2015) explore the GVC’s interconnectivity of industries and their flows at the global, regional, and local levels. Their findings are that industries are asymmetrically connected at the global level, which causes shocks to lead to fluctuation in the whole network, concluding that inter-relationships of industries identified at the cross-sectional country level are still at a regional level.

With a database from BACI-CEPII (Base pour l'Analyse du Commerce International-Centre d'Etudes Prospectives d'Informations Internationales), De Benedictis et al. (2013) uses centrality measures in a local and global sense to differentiate countries’ position in the general trade network as well in commodities such as Bananas, Cement, Movies, Oil, Footwear and Engines, concluding that the network for selected products is characterized by oligopolistic structure. More recently, Cingolani et al. (2017) used the electronic sector, motor vehicles, textiles, and apparel between 2007 and 2014, examining the effect of countries’ neighborhoods and the effect of third countries, allowing to detect clusters and hubs in the networks.

However, although the network concept has existed in the trade literature, its use has been limited in the field of the GVC. This delay is mainly because GVC literature focuses on how these value chains are formed, such as the snake and spider shapes (World Bank, Global Value Chain Development Report 2017), and not on the structure itself, its connectivity, and dependency relationships. Therefore, it is necessary to distinguish that the shape is not the same as the structure, and this is where the compatibility between the value-added flows on the GVC and the Network Science exists.

Based on the paper of Cingolani et al. (2017) and having as a primary reference the study of De Benedictis et al. (2013) and Cerina et al. (2015), I aim to go further with the network analysis. It is essential to highlight that these studies only provided the topological approach (structure) and the measure approaches, such as degree, density, and centralities. Because of this limitation, I would like to use a community detection algorithm, a frequently used tool in network science, to provide an extended version of interpreting and understanding the network structure. The reason to use this tool is that, contrary to the shape of the GVC that classifies a country according to the production stage, the network community uses link density and centralities to classify them.

The concept goes beyond grouping countries by their location. Instead, it groups them by the interconnectivity and the effect of third parties on the country *i* and *j* relationship. It is important because it classifies countries according to their structural position in the network, revealing hierarchical organization inside the community and the closest interdependency among the members. Its use is applied primarily in computational sciences, such as Lu et al. (2018), Yang and Le (2021), Yang et al. (2013), in biology, Vandeputte et al. (2017), or social science, Šubelj et al. (2016).

This study distinguishes transport equipment from others as, for the industry, core regions have been distinguished by different markets from the final demand, but not the inter-relations between the economies by the value-added contribution, except for Pavlínek (2021), who conducted an analysis for the automotive industry in the Eurozone. In addition, the industry is one of the manufacturing industries that capture more presence of Research and Development (R&D), management, and complex activities based on the labor market; all of them are essential characteristics in the GVC studies.

The transport equipment industry has peculiar characteristics to be considered a highly complex value chain. The industry is characterized as a mature industry that offers high-level products. Moreover, the value chain presents different degrees of fragmentation and technological capacities. That is, multinational companies (MNCs) seek places with the lower cost of the different stages of production. At the same time, in the main branch, they focus on developing the new technology to be added to the product. In other words, the industry added technical innovations to the existing products rather than developing new ones.

Schwabe (2020) provides a study focusing on German suppliers’ risks and strategies with the transition from combustion engines to electric engines in the transport equipment industry. He mentioned that lead firms tend to reallocate their production process close to the assembly lines due to the increased cost.

On a more disaggregated level, Fana and Villani (2022) decompose the automotive supply chain by analyzing the employment, the value-added, and the occupation structure for countries such as Germany, France, the United Kingdom, and Italy. They found that after the global financial crisis, the supply chains reorganized, being the Eastern Europe countries the ones who benefitted the more due to the strong position of Germany in the industry. Moreover, they emphasize that German car makers offshored the production of the intermediate components, kept the final assembly domestic, and dominated activities such as R&D. Grodzicki and Skrzypek (2020) found similar results using a panel-data ARDL model, concluding that Germany’s strong position in the GVCs is because the country can maintain high value-added inputs in its final goods. In contrast, Spain relies more on foreign suppliers.

Dussel Peters (2022) studied the relationship between the United States, China, and Latin America in the global auto parts chain. He highlights that China is gradually taking the place of Canada in the intra-NAFTA relationships as a result of its local production policy and exports oriented after joining the World Trade Organization. Moreover, he states that Mexico has benefited from the preferential tariffs originated from the agreement and from the recent forced local content in the automobile industry that the USMCA agreement demands. Nevertheless, the main limitation of this study is that even if it tries to explain the triangular relationship, it does from the aggregate value and not the value-added flows perspective.

From a geographical and knowledge creation perspective, Rodríguez-De la Fuente and Lampón (2020) studied the cases of the Mexican and Spain transport industries, mainly the automobile, which is the most representative of the industry. They concluded that the status of Mexico on the GVCs is characterized by a low added value and knowledge contents of production activities and cannot generate technology, and its value-added is mainly due to the North America Automobile production system. On the other side, Spain’s position is characterized by adding value and knowledge to the production activity; however, it is still in an intermediate position due to its dependency on the European production system. Crossa and Ebner (2020) got similar results for the Mexico case and Sancak (2021) for Mexican and Turkish suppliers.

The study by Lee et al. (2021) focuses on the automobile sector of China, Thailand, and Malaysia and compares their cases with the success of South Korea. They concluded that the success case of the upgrading case of China is due to the increase of the share of domestic value-added in their exports, labor productivity, and substantial investment in R&D. On the other hand, Thailand has focused on increasing their exports. However, the value-added content is from MNCs, mainly from Japan; as a result, Thailand has just the connector role in the industry. Finally, Malaysia still needs to increase its role in the GVCs due to the lack of competitiveness in the local markets; thus, the few existing local firms do not add value to their products nor focus on the exports side.

Nevertheless, none of the previous studies has conducted a global performance of the transport equipment industry, nor have they used the network approach to explain this phenomenon. To fill the gap in the GVC literature, this research aims to unravel the particular characteristics of the contribution of the value-added in the transport equipment industry network by addressing the following questions:

1. Is the transport equipment industry highly centralized from the value-added flows?
2. Which countries are part of the center and which are still far from joining the industry?
3. How can we measure the relevance of each country in the network?
4. Do communities exist in the industry, and what are their specific characteristics? Are they part of the same territory?

To answer the above questions, I used the UNCTAD-Eora Global Value Chain database, which contains information on the in- and out-flows of value-added of the industry, and this information is computed as a complex network system where countries are represented as nodes and value-added flows as edges.

One of the main contributions of this paper is that it analyzes the industry as a whole structure and provides information on the integration process in this network not only by region, but also identifying the leading economies that influence the movement of flows through their interactions. Moreover, by applying the various measures and answering the previous questions, the study clarifies the mixed results in the adopted policies that each country and regions have and how in/dependent on the production process they are. These measures confirm the existence of different governance structures in the industry across countries and regions that are attributable beyond a geographical factor. Finally, it contributes by expanding the use of the Network measures in the economic field related to the GVC studies.

The structure of this paper is as follows. Section 2 provides information about Data and the methodology. In Sect. 3, the main questions for this study are driven and provide the network analysis results, divided into four different approaches: the first approach consists of visual tools, the second approach applies measure tools, such as degree, clustering, density, which will reflect those countries with high out-degree dominate the supply of value-added in the industry, while high in-degree countries are the users. The third approach uses the centrality concept split into two major components, Eigen betweenness and Eigenvector centrality measures; they provide valuable information about the position of each country and its role in the industry. Finally, detecting communities, the fourth and last approach, provides information on highly related countries by grouping them according to the in–out-flows density. Section 4 concludes.

## Data sources and methodology

### Data

The empirical research uses data from the UNCTAD-Eora Global Value Chain Database (from now on, referred to as the Eora database). The data covers 189 different countries and regions time series related to critical indicators for the GVC as the foreign value added (FVA), domestic value-added (DVA), and indirect value added (DVX), which are generated from the EORA Multi-Region Input–Output tables (MRIOs) for the Transport Equipment Industry.

One of the advantages of using this database is the length of the time coverage, which uses the most recent information, 2017. In addition, it provides a broader panorama by including more countries and regions than the OECD TiVA data set. It is essential to highlight that, accordingly to the methodology used to calculate the flows proposed by Casella et al. (2019) for the Eora database, overall, the results for this dataset and the OECD confirm an alignment and consistent results.

Even though BACI-CEPII attempts to have the largest number of countries and time series and follows the reconciliation methodology purposed by Gualier and Zignago (2010) to reduce the number of missing values, this research aims to contribute to the results obtained by De Benedictis et al. (2013) by exploring the industry using the EORA database and providing an extension panorama of the network science studies related to trade.

To my understanding, the above studies have not addressed the Transport Equipment Industry issue from the context of the value-added contribution nor detecting communities according to the density of links between the members. In addition, none of the previous studies have used the Eora database, which gives this paper the novelty to be used for a different perspective and future reference for comparative analysis.

### Setting the network structure

Using the Eora database, only 129 countries provide information about the Transport Equipment Industry, creating 16,461 edges. However, to better understand the network characteristics, I applied a cut set to provide more specific results in the industry. This cut set uses the “total network average flow” benchmark to select only the top flows in the industry. As a result, the final selection covers 62 countries (nodes) and 689 edges, accounting for 97% of the total Transport Equipment Industry value-added flows.

### Methodology

As the proper methodology in the Eora database says, the derivation of the value-added trade from the MRIO tables has to be established by using the standard IO analysis, and this model can be expressed as:

1 $$X = AX + Y,$$

where *X* is the vector of the total outputs by countries; *A* reflects the vector of intermediate uses, the inter-industrial matrix between all economies measured per unit output; and *Y* reflects the final demand. The relationships of countries in (1) can be expressed in terms of the MRIO framework as:

2 $$X = \left( {I - A} \right)^{ - 1} Y = LY,$$

where $L = \left( {I - A} \right)^{ - 1}$ is the Leontief inverse matrix which provides the information of direct and indirect outputs to satisfy one unit of the final demand.

Therefore, based on Casella et al. (2019) methodology, this can be transferred as well to the value-added trade framework between countries and can be expressed as:

3 $$F = \left( {\begin{array}{*{20}c} {F^{11} } & \ldots & {F^{1N} } \\ \vdots & \ddots & \vdots \\ {F^{N1} } & \ldots & {F^{NN} } \\ \end{array} } \right) = \left( {\begin{array}{*{20}c} {V^{1} } & 0 & 0 \\ 0 & \ddots & 0 \\ 0 & 0 & {V^{N} } \\ \end{array} } \right)\left( {\begin{array}{*{20}c} {L^{11} } & \ldots & {L^{1N} } \\ \vdots & \ddots & \vdots \\ {L^{N1} } & \ldots & {L^{NN} } \\ \end{array} } \right)\left( {\begin{array}{*{20}c} {E^{1} } & 0 & 0 \\ 0 & \ddots & 0 \\ 0 & 0 & {E^{N} } \\ \end{array} } \right) ,$$

where *F* shows the Transport Equipment flows between countries *r* (*r* = 1,…*N*) and countries *s* (*s* = 1, …, *N*). *V* represents the value/added share, while *E* represents the exports. Hence, this matrix describes the value-added contained in the transport equipment industry.

The value-added contribution as a supplier in the transport equipment industry is defined as the total sum of exports of countries *r* to meet the final demand of all other regions and can be described as:

4 $$F_{r}^{\exp } = \sum \limits_{s \ne r}^{N} F_{rs} .$$

The value-added contribution as a user in the transport equipment industry is defined as the total sum of imports of countries *r* from all other regions, handled by the final demand of countries r and is indicated by:

5 $$F_{r}^{{{\text{imp}}}} = \sum \limits_{s \ne r}^{N} F_{sr} .$$

Therefore, the total balance of the value-added contribution is obtained as follows:

6 $$F_{r}^{{{\text{net}}}} = F_{r}^{\exp } - F_{r}^{{{\text{imp}}}} .$$

Hence, countries *r* become net suppliers when the balance is positive; otherwise, they are users.

### Network indicators

The value-added contribution in the transport equipment industry flows can be presented as a complex network structure: the countries that participate in both the supply and the user side are the nodes, while the flows among them are the edges. It can be characterized in a binary form (undirected) or directed. The difference is that the first only reflects the existence of a link between the nodes, while the second reflects the weight of the link, that is, the total flows in each link and if it is an in or outgoing link.

This research follows the network calculations proposed by Barabási (2016) and uses fundamental properties of nodes involved in the network, such as its degree, clustering, centrality, and communities.

#### Degree and degree distribution

In directed networks like the present one, the distinction between incoming and outcoming flows is necessary. Therefore, in-degree and out-degree measures would be used to calculate the total degree of the node. This representation is as follows:

7 $$k_{i}^{{{\text{in}}}} = \sum \nolimits_{j = 1}^{N} g_{ij} ,$$

8 $$k_{i}^{{{\text{out}}}} = \sum \nolimits_{j = 1}^{N} g_{ji} ,$$

9 $$k_{i} = k_{i}^{{{\text{in}}}} + k_{i}^{{{\text{out}}}} ,$$

where $g_{ij}$ is a dummy variable that denotes whether there is a contribution in value-added flows in the transport industry from economy *i* to economy *j*, *N* represents the total number of economies in the network, *k* represents the degree, while $k_{i}^{{{\text{in}}}}$ and $k_{i}^{{{\text{out}}}}$ indicate the in-degree and out-degree, respectively, and $k_{i} { }$ is the total degree of the node. In the case of this study, a weighted directed network is used, which means the edges that connect two economies are weighted in portion to the flows between them.

The degree distribution measure provides the probability that a randomly selected node has a degree *k*. Since we want to know the probabilities and analyze the scale-free structure of the network, the normalized probability distribution is as follows:

10 $$\sum \limits_{k = 1}^{\infty } p_{k} = 1,$$

where $p_{k} = n_{k} /n$, and $n_{k}$ is the number of economies with the same degree *k*. That is, $p_{k}$ indicates the probability that a given node has degree *k* in the network. Equation 10 provides the network’s cumulative degree distribution, that is, the sum of all probabilities of all nodes with *k* degrees, equivalent to 1 for the normalized probability distribution.

In addition, the network is scale-free if a power-law distribution well fits its degree distribution, that is, $p\left( k \right) \propto k^{ - \gamma }$, meaning that few countries with larger links and high centralities exist and many others with few links and low centralities. Important it is to say that networks have a classification, such as random networks and scale-free. For more details consult Barabási (2016).

#### Paths and distance

Gravity models suggest that physical distance is a significant component that affects trade between economies, saying that the closer they are, the more trade between them. In the context of a complex network, distance is a more challenging concept. Since the network framework lacks a concept of physical distance, this is replaced by path length. A *path* is a route that runs along with the links of the network and measures the number of economies (for this study) that need to pass through between two economies for a value-added contribution trade relationship in the transport equipment industry.

There exist some properties related to path and distance. The shortest path between nodes *i* and *j* is the one with the fewest edges. In diverse literature, the concept is often called the distance between node *i* and *j*, denoted by $d_{ij}$. In undirected networks, where it does not matter the direction of the flows, the distance between *i* and *j* is the same; however, this does not apply for directed networks, where the existence of a path from node *i* to node *j* does not assure the existence of a path from *j* to *i*.

What concerns the most for this study is, on average, how many economies a randomly selected country needs to pass through. The average path length is denoted by:

11 $$\left\langle d \right\rangle = \frac{1}{{N\left( {N - 1} \right)}} \sum \limits_{i,j = 1,N;i \ne j} d_{ij} .$$

#### Clustering coefficient and density

Clustering coefficient expresses the degree to which the neighbors of a given node relate to each other. For our specific network, it will measure if trade relations by the added-value contribution exist between the trading partners in the whole network and has to be calculated in the average over all nodes, which is:

12 $$\left\langle C \right\rangle = \frac{1}{N} \sum \limits_{i = 1}^{N} c_{i} ,$$

where *N* is the nodes (economies) in the network, and $c_{i} = \frac{{e_{i} }}{{k_{i} \left( {k_{i} - 1} \right)}}$ represents the clustering coefficient of a specific node, obtained by the number of links $e_{i}$, between the $k_{i}$ neighbors of node *i*. Therefore, the more densely interconnected the network, the higher the clustering coefficient.

A complete network is when all nodes are connected, reflecting the complexity of reciprocity among the nodes, in this case, the economies. If links connect all nodes, we said the network is totally dense, while the lower range, the less dense the network is. Density can be described as follows:

13 $$D = \frac{2e}{{N\left( {N - 1} \right)}},$$

where $e$ is the current links in the network, *D* ranges between 0 and 1. Real networks expect to have a coefficient far less than 1.

#### Centrality

An economy can play an important role as a user and supplier of value-added flows in the network as it acts as a catalyzer for transferring this value-added contribution. Such importance for connectivity can be measured by the betweenness centrality and the eigenvector centrality.

Betweenness centrality indicates how important a node is in terms of connecting other nodes (De Benedictis et al. 2013) and is obtained by:

14 $$b_{k} = \sum \limits_{i = 1}^{n} \sum \limits_{j = 1}^{n} \sigma_{ij} \left( k \right)/\sigma_{ij} ,$$

where $\sigma_{ij}$ is the number of shortest paths between economy *i* and economy *j*, $\sigma_{ij} \left( k \right)$ is the number of shortest paths between economy *i* and *j* that pass-by economy *k* (Liu et al. 2022).

Eigenvector centrality calculates the node influence by evaluating the importance of its neighbors; as De Benedictis et al. (2013) pointed out, what matters is the centrality of the linked countries to a specific node and not the node’s centrality itself. Hence, a country is influential in the network by being associated with the countries with large value-added contribution flows. The measure is defined as:

15 $$v_{i} = \lambda^{ - 1} \sum \limits_{j = 1}^{{n_{i} }} g_{ij} v_{j} ,$$

where $\lambda$ and $v_{j}$ correspond to the largest eigenvalue and the associated eigenvector.

The advantages of using this measure are that it will provide the importance of the country itself, the importance of its neighbors, and the effects of the third countries on the selected country.

#### Communities

A group of nodes that tend to have a higher plausibility of connecting to each other is called a community in network science. One of the most used algorithms to detect community groups in network science was proposed by Girvan and Newman (2002), which consists of the use of the link betweenness as a centrality measure in order to detect the shortest path between the nodes and cut one by one the less significative edges that connect nodes to generate the communities. It also uses the modularity function to select the optimal cut to divide into groups. The algorithm function is as follows:

16 $$Q = \frac{1}{2m} \sum \limits_{i = 1}^{N} \sum \limits_{j = 1}^{N} \left[ {w_{ij} - \frac{{z_{i} z_{j} }}{2m}} \right]\delta \left( {c_{i} ,c_{j} } \right),$$

where $w_{ij} = F_{ij} + F_{ji}$ is the total amount of value-added flows between country *i* and country *j*; $z_{i} = \sum \limits_{j = 1}^{n} w_{ij}$ is the sum of value-added flows attached to economy *i*, the indicators *c* represents the community to which economy is assigned; $\delta \left( {c_{i} ,c_{j} } \right)$ is a function that takes the value of 1 if $c_{i} = c_{j}$ and 0 otherwise; and $m = \sum \limits_{i = 1}^{n} \sum \limits_{j = 1}^{n} w_{ij} /2$.

Besides community detection, the algorithm detects the cliques inside the whole network. Luce and Perry (1949) defined a *community* as a group of individuals whose members know each other; consequently, a clique is a complete subgraph with maximal link density. This type of graph allows detecting the hierarchical clustering in a graph, suggesting which nodes are more likely to join and lead the industry.

#### Complementary measures

There are some measures that provide information in how the structure of the network is. These measures are more related with the connectivity of the network and allow us provide complementary information from the measures above.

According to Newman (2003), assortativity refers to the increase or reduction in the probability of connecting two nodes based on their correlation degree. With this measure, it is possible to check if countries are likely to trade with those with similar values (if the value negative) or if countries tend to trade with those with different values (positive value). Assortativity is calculated as follows:

17 $$\Omega = \frac{{ \sum \nolimits_{jk} jk\left( {e_{jk} - q_{j} q_{k} } \right)}}{{\sigma_{q}^{2} }},$$

where $q_{k}$ is the distribution of the remaining degree, $e_{jk}$ represents the jointly probability distribution of the remaining degrees of the two nodes, $e_{i,j}$ is the fraction of edges connecting nodes of type *i* and *j* while $\sigma_{q}^{2}$ is the standard deviation of *q*, in which q is the sum of all $e_{i,j}$ in both ‘in’ and ‘out’ flows in a directed network.

In addition, reciprocity is most commonly defined as the probability that exists mutual connections of a directed link between existing nodes, that is, that the counterpart of a node also includes a link for the selected node. This measure is calculated as follows:

18 $$R = \frac{{ \sum \nolimits_{ij} \left( {A \cdot A^{\prime}} \right)_{ij} }}{{ \sum \nolimits_{ij} A_{ij} }},$$

where $A \cdot A^{\prime}$ is the element-wise product of a matrix $A$ and its transpose, which in this case, reflects the contribution of value-added.

Following Freeman (1979), centralization is a general method for calculating a graph-level centrality score based on node-level centrality measure, which is a complement of the simple degree measure. This score reflects the degree in which links spreads throughout the network, proving information of a possible existence of cluster if the score is high. Centralization is obtained as follows:

19 $${\mathbb{C}} = \frac{{\sum\nolimits_{n} {\left( {\mathop {\max }\limits_{w} {\mathbb{c}}_{w} - {\mathbb{c}}_{n} } \right)} }}{{\left[ {\left( {N - 1} \right)\left( {N - 2} \right)} \right]}},$$

where ${\mathbb{c}}_{n}$ is the centrality of node *n* and $\mathop {\max }\limits_{w} {\mathbb{c}}_{w}$ represents the maximum value in the network. The graph centrality score can be normalized by dividing by the maximum theoretical score for a graph with the same number of edges as the graph under study, in this scare, the transport equipment industry network.

## Main results

This section provides general details on the value-added network in the transport equipment industry and analyses its structure on both sides, the users and suppliers, during 2017.

### First approach: visualization

**Fig. 1:**
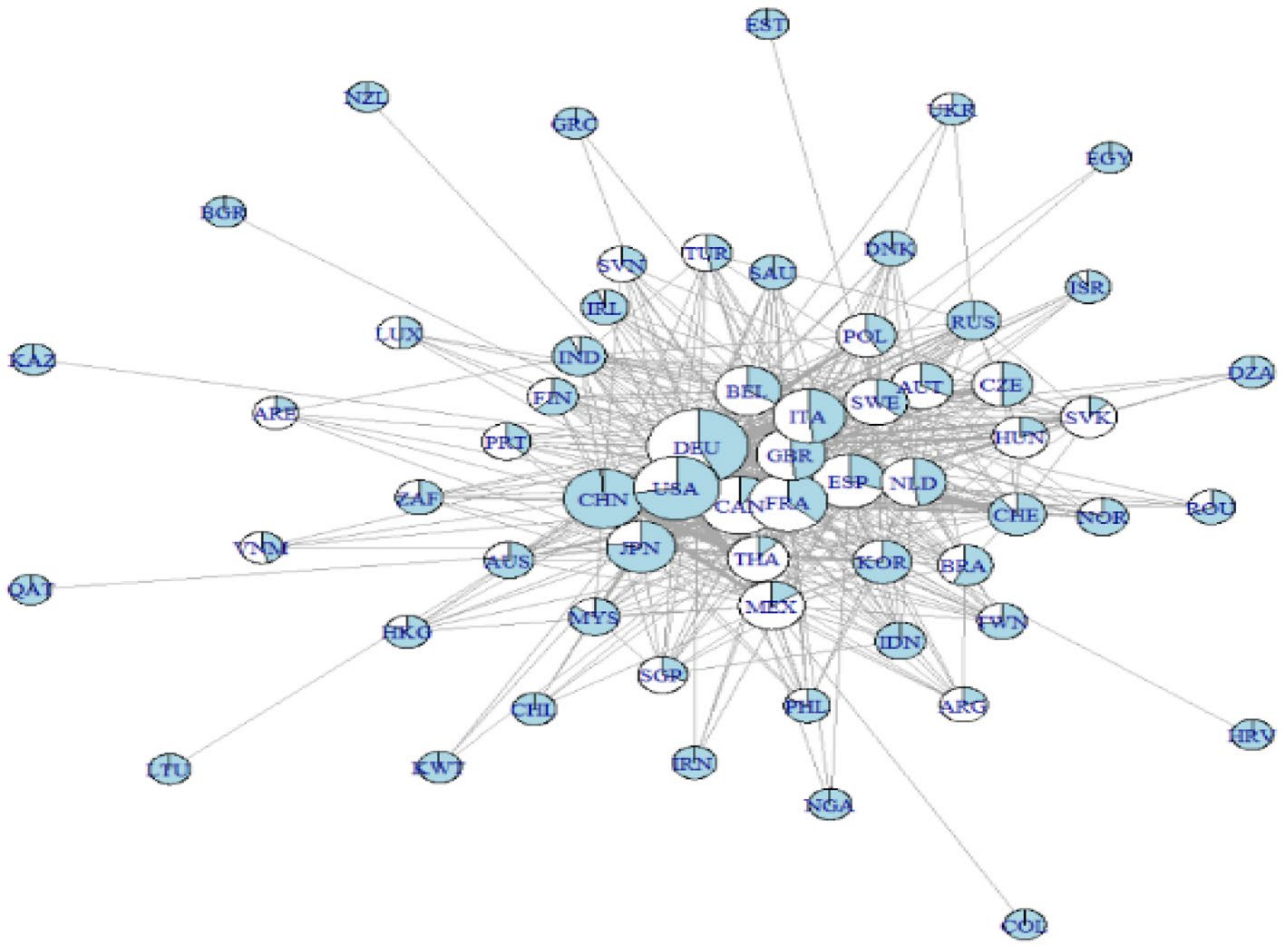
Value-added contribution in Transport Equipment Network. The node area reflects the total flows of value-added in or out of the industry, with relative proportion of in- and out-flows indicated in blue and white, respectively. Edges’ width reflects the volume of flows from the origin and destination. The Large Graph layout was used to produce this graph (Source: Own elaboration with EORA data)

Figure 1 provides the network structure. Each country represents a node, and the area reflects the total flow of value-added in or out, with the relative proportion of in-flows and out-flows indicated in white and blue, respectively. In addition, the width of the links reflects the volume of the flows regarding the origin and destination.

As Amador and Cabral (2016) pointed out, larger economies tend to have a bigger size and are located in the center of the network, mainly because they contribute more to the value-added and smaller countries tend to be placed outside the center. The value-added has been concentrated primarily in developed countries such as the United States, Germany, France, Italy, and Japan, with a few developing countries such as China, Thailand, Mexico, Poland, and Brazil.

The result of these countries’ position is as expected. Mainly due to the contribution of the value-added from automotive manufacturers to the transport equipment industry, in which firms such as Daimler (Germany), General Motors (United States), Renault (France), Fiat (Italy), Nissan (Japan) and Toyota (Japan) have higher market participation worldwide. In addition, these firms have filial in Poland, Mexico, Brazil, and Thailand. In the case of China, the strong local demand has helped the country to create value through their national firms, such as SAIC Motors, Dongfeng, and Changan Automobile, among others. Performance of different firms was conducted by Ferreira et al. (2021), highlighting the role of Japanese firms in the industry to have greater dominance with alliances such as the Nissan–Renault (Japan–Italy) and Ford–Mazda (Japan–United States). On the side of the aircraft, American companies dominate the industry with some relevant competition from Russia, France, Netherlands, Italy and Japan.

European countries in the center have a more significant proportion of out-flows than in-flows, meaning that the value-added they generate is smaller than they receive. As Pavlínek (2021) demonstrates for the European automotive industry, this result is expected due to the integration process these countries already have. Moreover, according to the European business fact and figures from Eurostat (2018), the manufacture of transport equipment within the European Union concentrates more than 70% of the value added of the industry in the motor and vehicles sectors, followed by Aircraft and Spacecraft with no more than 15%, Ships and boats 6% and the rest between railway equipment and miscellaneous transport equipment (European Business: Facts and figures—2009 edition); therefore it is not a surprise to see in the center of the network countries such as Germany, Italy, France, Spain, and in less extend the United Kingdom and Sweden, which are the leading producers in the region for the automobile industry.

Contrary, the United States’ in-flows proportion is explained by the larger out-flows that Canada and Mexico have due to NAFTA, which can be seen with the width of the links between them, reflecting the importance of the industry for this region. The same applies to Japan, as the country has been investing in Mexico in the automotive industry.

For countries such as Thailand, South Korea, Mexico, Brazil, Poland, and the Czech Republic, whose transport equipment industry is essential, their position on the network is such that it reflects their degree of integration in the value chains as users and suppliers of value-added, especially since its trade relations are mainly with countries at the center. However, as we will show later with the centrality measures (Sect. 3.3), the position of these countries, except for South Korea, is because of their relatively lower cost, their geographical proximity to the centers, optimized transportation options, and enough labor force.

Some caution is necessary when reading the value-added contribution for countries further away from the center. As Fig. 1 shows, these countries’ flows are out-flows, except for Ukraine and Romaine, but these flows are relatively smaller than countries in the center or close to them, suggesting these countries are still behind in joining the GVC on the industry.

### Second approach: measures

Density describes the portion of possible connections in a network that are actual connections. Table 1 describes the network statistics. For the contribution on the value-added in the transport equipment, by applying Eq. (13), density is 0.1821, meaning that from the total possible edges, the network consists of only 18.21%, even though these flows contribute 97% of the total flows. In other words, the existing nodes and their flows account for almost the totality of the industry, which lead us to infer a high degree of concentration; therefore, a possible cluster.

**Table 1:** Network statistics.

Density	0.1821
Reciprocity	0.5341
Centralization	0.5896
Assortativity	− 0.5336
Clustering	0.76
Average path length	1.8

Source: Own elaboration with EORA data

Reciprocity expresses the level of two-way ties, where two economies contribute and use value-added from one another. Using Eq. (18), the network has a 0.5341 reciprocity coefficient, meaning that more than half of the edges show reciprocity between them. Given that the coefficient is above 0.5, we infer a high degree of integration of the transportation equipment industry, mainly due to the regionalization and fragmentation of the production process that causes more and more countries and regions to become involved in the value creation chains.

**Fig. 2:**
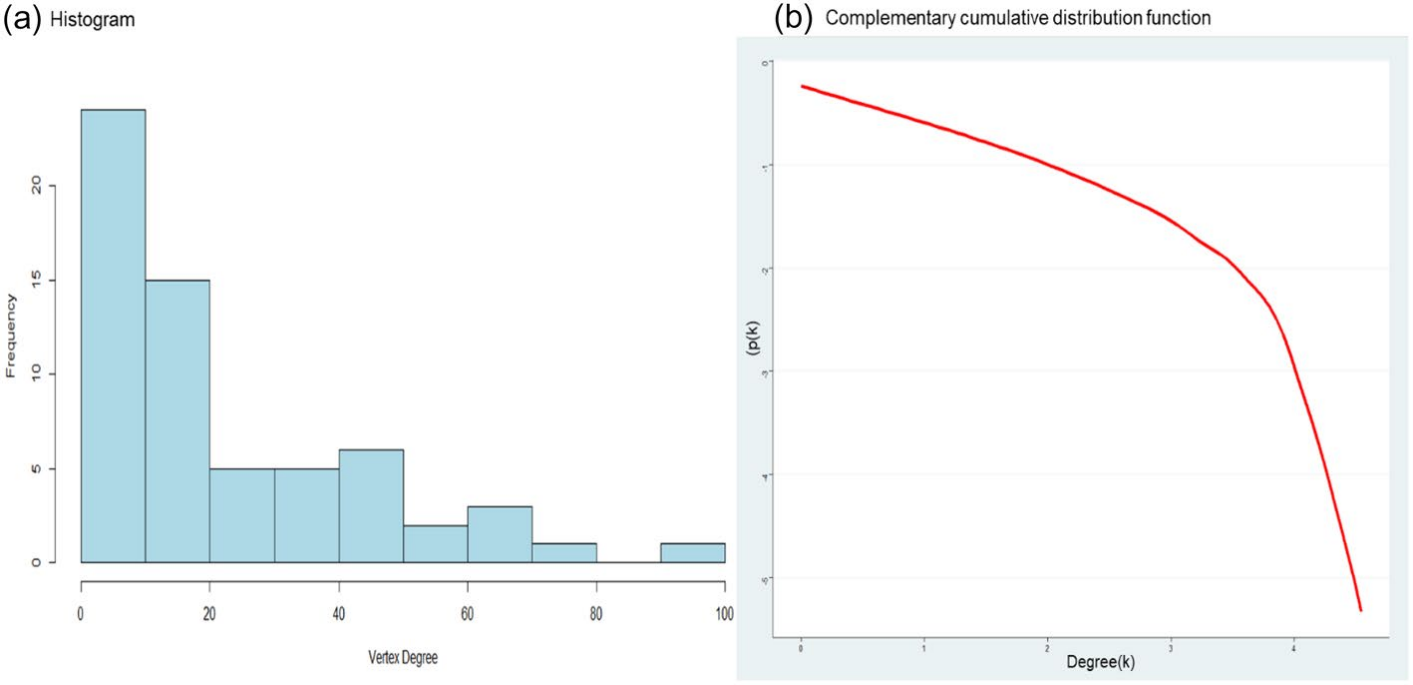
Degree distribution of the Transport Equipment Industry. **a** Provides the histogram of the degree distribution. The Horizontal axis measures the number of links each country has and the frequency on the vertical axis reflects the number of countries with that degree. **b** Provides the complementary cumulative distribution in its log–log scale (Source: Own elaboration with EORA data)

As Fig. 2a shows, more countries with a lower degree exist. Panel (b) suggests that the network follows a power-law distribution. Few economies can be called “hubs” in the network, meaning that few economies concentrate the higher values on the network due to a large number of connections, as approximately 20% of the total links are in the hands of those countries with a degree equal or higher than 40.

Table 2 provides the information on the total degree by country and its ranking. These results are obtained by applying Eq. (9). Only 13 countries have a degree higher than 40, reinforcing the previous statement. Refer to Table 3 in Appendix for the country list.

**Table 2:** Network degree by country.

Total (inflow and outflows)					
DEU	93	SVK	25	ROU	7
FRA	71	IND	23	ARE	7
USA	69	RUS	20	CHL	6
ITA	67	FIN	18	LUX	6
ESP	63	TUR	18	DZA	4
GBR	60	MYS	17	IRN	4
JPN	56	TWN	17	VNM	4
BEL	49	NOR	16	KWT	3
CAN	49	PRT	16	NGA	3
NLD	46	IDN	15	UKR	3
SWE	46	SGP	15	EGY	2
AUT	43	AUS	14	GRC	2
CHN	42	PHL	12	BGR	1
THA	38	SVN	12	COL	1
MEX	36	ZAF	12	HRV	1
CZE	34	ARG	11	EST	1
KOR	34	IRL	11	KAZ	1
POL	33	DNK	10	LTU	1
BRA	30	SAU	10	NZL	1
HUN	28	HKG	7	QAT	1
CHE	26	ISR	7		

Source: Own elaboration

**Table 3:** Country codes and names.

DZA	Algeria	LTU	Lithuania
ARG	Argentina	LUX	Luxembourg
AUS	Australia	MYS	Malaysia
AUT	Austria	MEX	Mexico
BEL	Belgium	NLD	Netherlands
BRA	Brazil	NZL	New Zealand
BGR	Bulgaria	NGA	Nigeria
CAN	Canada	NOR	Norway
CHL	Chile	PHL	Philippines
CHN	China	POL	Poland
COL	Colombia	PRT	Portugal
HRV	Croatia	QAT	Qatar
CZE	Czech Republic	KOR	South Korea
DNK	Denmark	ROU	Romania
EGY	Egypt	RUS	Russia
EST	Estonia	SAU	Saudi Arabia
FIN	Finland	SGP	Singapore
FRA	France	SVK	Slovakia
DEU	Germany	SVN	Slovenia
GRC	Greece	ZAF	South Africa
HKG	Hong Kong	ESP	Spain
HUN	Hungary	SWE	Sweden
IND	India	CHE	Switzerland
IDN	Indonesia	TWN	Taiwan
IRN	Iran	THA	Thailand
IRL	Ireland	TUR	Turkey
ISR	Israel	UKR	Ukraine
ITA	Italy	ARE	UAE
JPN	Japan	GBR	UK
KAZ	Kazakhstan	USA	USA
KWT	Kuwait	VNM	Viet Nam

Source: Own elaboration with the EORA Country Codes

Germany has more connections, followed by France, the United States, Italy, and Spain. Particular interest is that top countries belong to Europe except for the United States. These results suggest that the value-added contribution in the industry is dominated by advanced economies, as Fig. 1 shows. However, each country’s degree only reflects the number of links each country has, not their influence over the entire network. Therefore, a more advanced analysis is necessary.

Centralization is a complement to the simple degree measure. It provides the distribution of these degree centrality scores. Borgatti et al. (2018) note that low centralization scores suggest that trade ties are spread uniformly throughout the network. In other words, if the score is high, the flows are concentrated in a small pair of countries, while a low score suggests no existence of a cluster. The centralization score is 0.5896 calculated using Eq. (19); the value-added flows tend to be concentrated in a small set of countries, potentially getting towards a hierarchical network structure.

Assortativity refers to the increase or reduction in the probability of connecting two nodes based on their correlation degree. A positivity coefficient would indicate that low degree countries connect with high degree countries, whereas a negative suggests that countries are likely to interact with those with similar degree centrality scores. The − 0.5336 coefficient shows that ties between the nodes tend to connect with similar ones, which was obtained from Eq. (17).

Applying Eq. (12), the clustering coefficient is 0.76, indicating that three-quarters of the neighbors of a selected node may become neighbors of other nodes or that a random node may be connected with the hubs. A Supporting measure is the average path length, with a coefficient of 1.8. On average, most proximately connected economies indirectly associated with other nodes through their neighbors in only 1.8 steps, accordingly with Eq. (11).

### Third approach: centralities

Betweenness centrality and eigenvector centrality are the most valuable tools to analyze the importance and influence of specific nodes. The former indicates how well situated a node is in terms of the path that it lies on, while the latter provides information about the centralities of countries that are surrounded and linked by; in other words, it reflects the importance of the countries from which the node is connected and the influence by third countries.

**Fig. 3:**
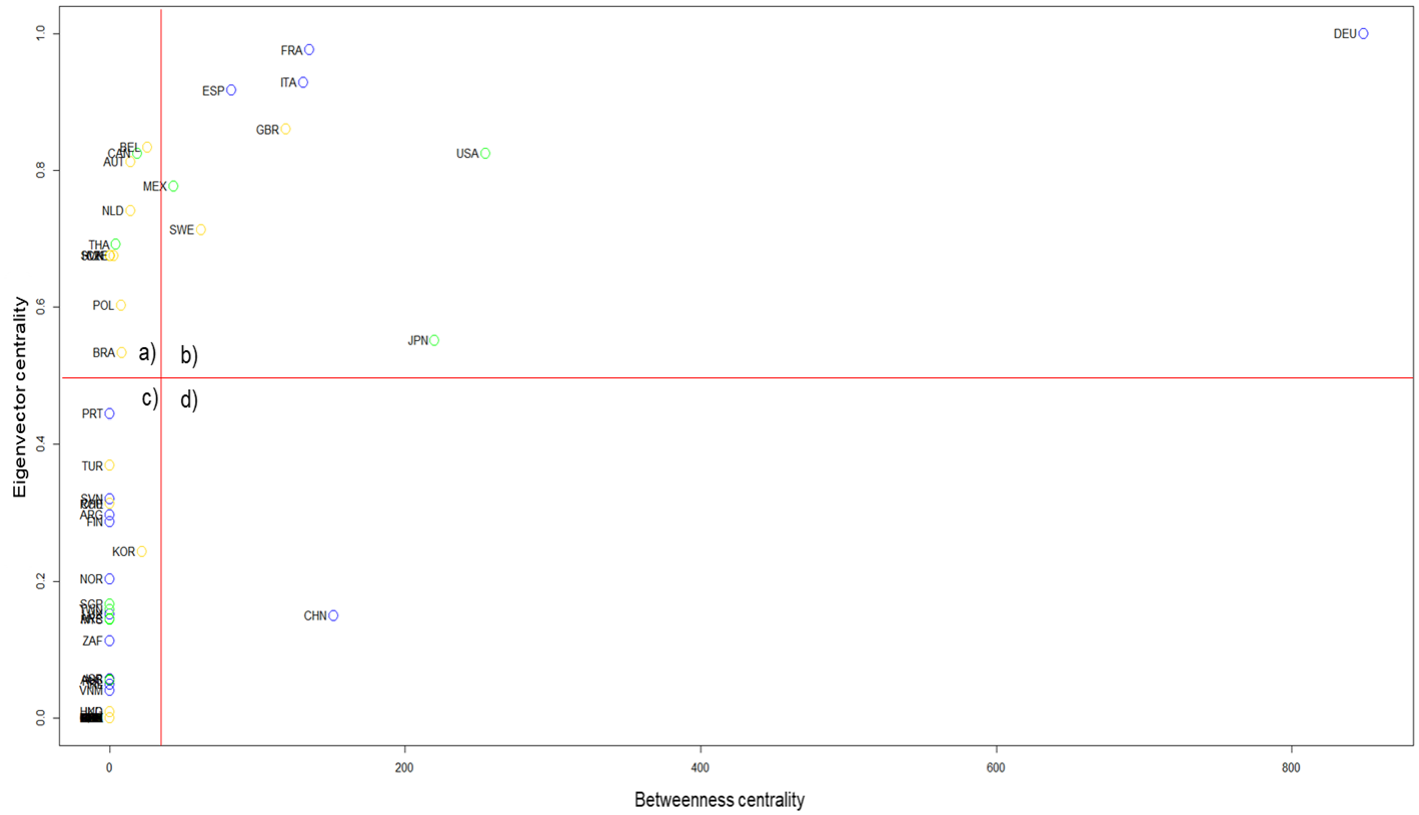
Centrality measures. The graph shows the relation of the betweenness centrality and the eigenvector centrality by country. **a** Reveals high low betweenness centrality and high eigenvector centrality. **b** Provides both high betweenness centrality and high eigenvector centrality. **c** Shows both low betweenness centrality and low eigenvector centrality. **d** Inform high betweenness centrality but low eigenvector centrality. The division of betweenness centrality relies on the mean for all countries, while eigenvector centrality takes 0.5 as the division point (Source: Own elaboration with EORA data)

Related to interconnectivity, measured by the betweenness centrality in Eq. (14), and reflected in Fig. 3, Germany, the United States, Japan, China, and France are the countries with the best results. On the other hand, countries with higher eigenvector centrality, and obtained from Eq. (15) are Germany, France, Italy, Spain, and the United Kingdom. Both results place Germany as the most crucial country in the network. In addition, the position of France in the network is essential as it ranks in the top five economies. Eigenvector centrality suggests that from the point of view of the neighbors, Europe tends to be the center of the industry, indicating that the value-added contribution is still at a regional level.

Within European Union, Germany concentrates 30.4% of the value added in the motor trade, followed by France, Italy, Spain, and the Netherlands with 15.8%, 10.1%, 8.0%, and 5.5%, respectively, in 2018 (European Commission, Eurostat 2021). On the other hand, Germany leads the employment in the industry with 25.3%, alongside France (12.0%), Italy (11.0%), Spain (8.5%), and Poland (8.5%). Hence, the position of Germany as the creator of the value-added in the motors trade is non-refutable.

Figure 3 divides the position of countries into four different sections. Panel (a) includes countries with low betweenness centrality but high eigenvector centrality; that is, the importance of these countries is more from the side of the effect of third countries and their neighbors than their proper location on the network. This panel includes Belgium, Canada, Netherlands, Thailand, Czech Republic, Slovakia, Hungary, Poland, and Brazil.

For the European Union, Belgium and Slovakia figures as the top five countries with the largest share of distribution of the value-added in the motor trade, with 14.6% and 14.2%, whereas Hungary and Poland have less average personnel cost in the motor trade from the same region (European Commission, Eurostat 2021). Therefore, its position in Fig. 3a is the reflection of their policies to concentrate value-added from third countries instead of creating themselves. Thailand abandoned its policy of local content in the transport industry after China joined the WTO to be enough competitive in the region. As a result of this policy, lower tariffs attract diverse MNCs and since then, Thailand has focused its trade policy in exports oriented instead of continue creating value for their local firms (Lee et al. 2021). Hence, the dependency of Thailand in third parties in the network is evident.

On the other hand, panel (d) provides high betweenness centrality but low eigenvector centrality. Only China is inside this panel, which suggests China’s importance is because of its position in the network and not properly because of its neighbors. Lee et al. (2021) already pointed out that the Chinese environment in the transport equipment industry favors adding value locally and is self-sufficient in supplying its market. Due to the country’s large population, getting a labor force with sufficient skills has not been a problem. In recent years, China has upgraded its value chain from being a simple assembler to creating its products to investing in and developing new technology through R&D.

Even China has managed to locate its products with medium–high value content (auto parts and components) abroad, mainly in the United States; this has not led to total dependence on the global market, which is why China’s industry is highly resilient. Thus, China is placed in panel (b) where its position in the structure does not correspond to the influence of other countries’ relations.

Panel (c) reflects countries with low coefficients in both measures; they have no advantageous position in the network, nor are they important to their surrounding countries’ effect. Surprisingly, South Korea is in this panel even though its position is close to joining China in panel b. The same situation is seen with Portugal, but close to panel a. Tukey, Argentine, Finland, and Slovenia are other countries inside this panel.

Possible explanations for South Korea’s position are that even though Korean transport equipment is representative in the manufacturing sector (with companies such as Hyundai and Kia motors), the increasing share of services activities in the country-oriented to exports has changed the composition of the value-added and GDP in the country. In addition, in 2017, South Korea experienced a decline in motor vehicle part production due to weak global demand and increased production costs. Furthermore, the non-favorable environment in the country created that General Motors started to consider closing one of its facilities, and eventually, 1 year later happened.

Finally, panel (b) provides countries well located in both measures because of their proper location in the network and the importance of their neighbors. The panel reveals the leader position of Germany in the industry, followed by advanced countries such as Japan, the United States, France, Italy, Spain, the United Kingdom, Sweden, and Mexico, the only developing country.

In the manufacturing industry of Mexico, 50% of the total came from the automobile industry, which represents approximately 18% of the GDP of the country, according to the National Institute of Statistics and Geography of Mexico. This high participation is the result of Mexico’s foreign trade policy, which has successfully attracted FDI from Japan, Germany, and South Korea, and also as the NAFTA integration. Mexico has created a cluster in the “Bajío” zone, with preferential tariffs, lower costs, and a labor force. However, Mexico has failed to absorb the technology and transfer it to the local producers, which creates that the value added originated in Mexico has not increased. Therefore, even though Mexico is in panel (d), its importance in the industry is because the country offers a suitable environment for MNCs to reallocate their production, allowing the country to satisfy the international demand.

Results proved that advanced European countries lead the industry by the added-value flows, and as a result of the United States and Japan’s investment in Mexico in the industry, this last country has gained importance.

Notwithstanding, as De Benedictis et al. (2013) mentioned, centralities measures must be read carefully. In one sense reflects the significance of the country as a central role or, on the other hand, the severe dependence on significant economies, such as the case of Mexico being part of panel b in Fig. 3.

One way to confirm these results is by finding the largest cliques in the network, which helps detect the countries that compound a subgraph; that is, all countries have connections between them.

**Fig. 4:**
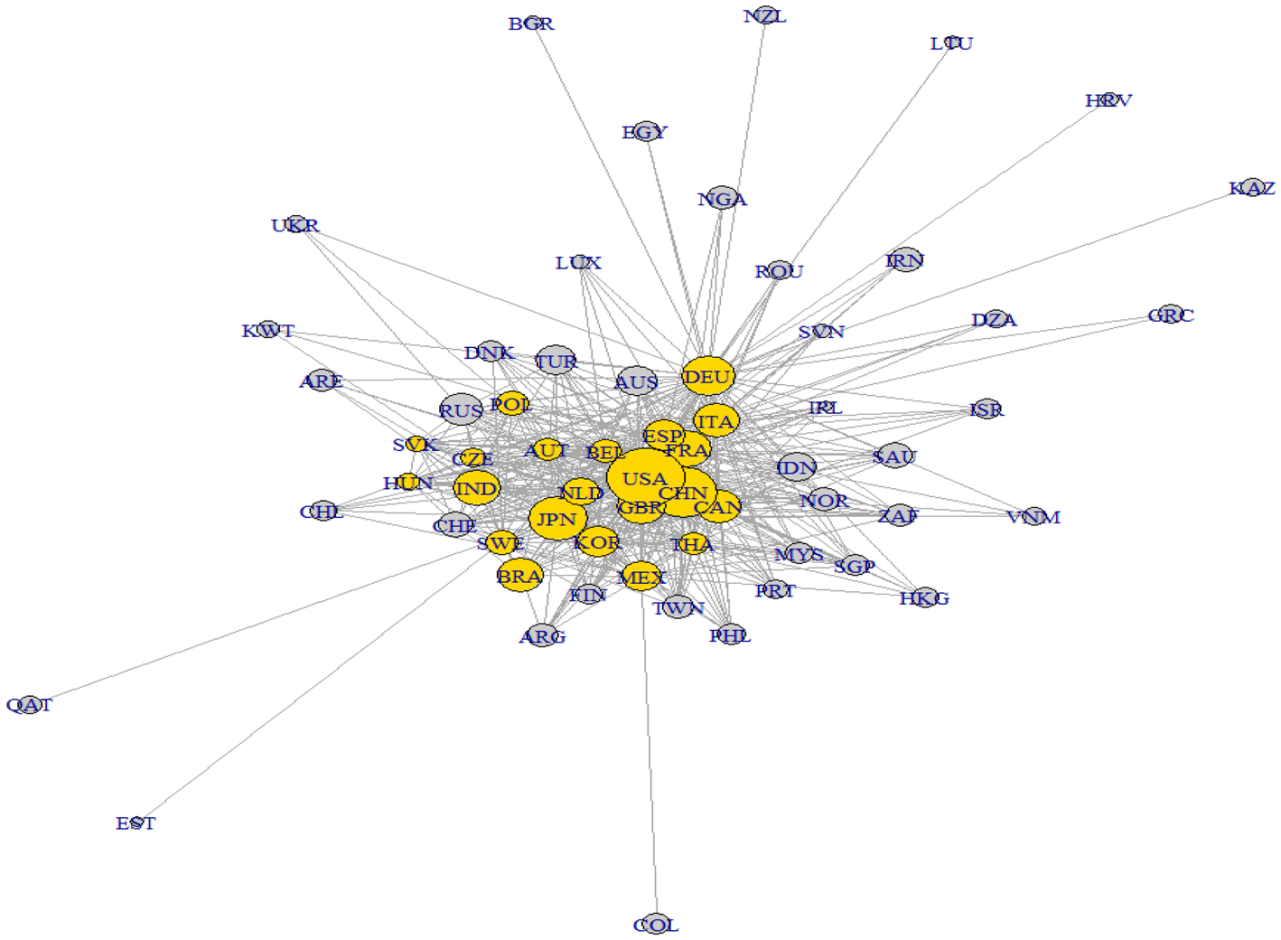
Largest clique. Color represents the countries that are inside the largest clique. The Fruchterman–Reingold layout was used to produce this graph (Source: Own elaboration with EORA data)

Figure 4 shows the two largest cliques, consisting of 18 countries. Both cliques share 14 members; the rest four differs. The countries that are inside both subgraphs are Germany, France, Spain, Italy, United States, United Kingdom, Belgium, Japan, China, Austria, Sweden, Netherlands, India, and South Korea, which compounds the central cluster with two different patterns which include (a) Slovakia, Czech Republic, Hungary and Poland (East Europe), and (b) Canada, Mexico, Brazil (American), and Thailand.

However, it is still not clear which countries relate with others, which is the main scope of the following subsection.

### Fourth approach: community detection

Detecting a community helps identify groups of countries that are densely interconnected but sparsely connected with others. The algorithm used in the research identifies the nodes to be part of only one community because it relies on the hypothesis that the partition offers the best community structure. As a result, communities are unique.

Contrary to geographical classification such as core and periphery, community detection would reveal mutual preferences on the basis that one economy can use and aggregate another’s country value-added more easily with those who are part of the same community than those who belong to other communities and are also further from them.

**Fig. 5:**
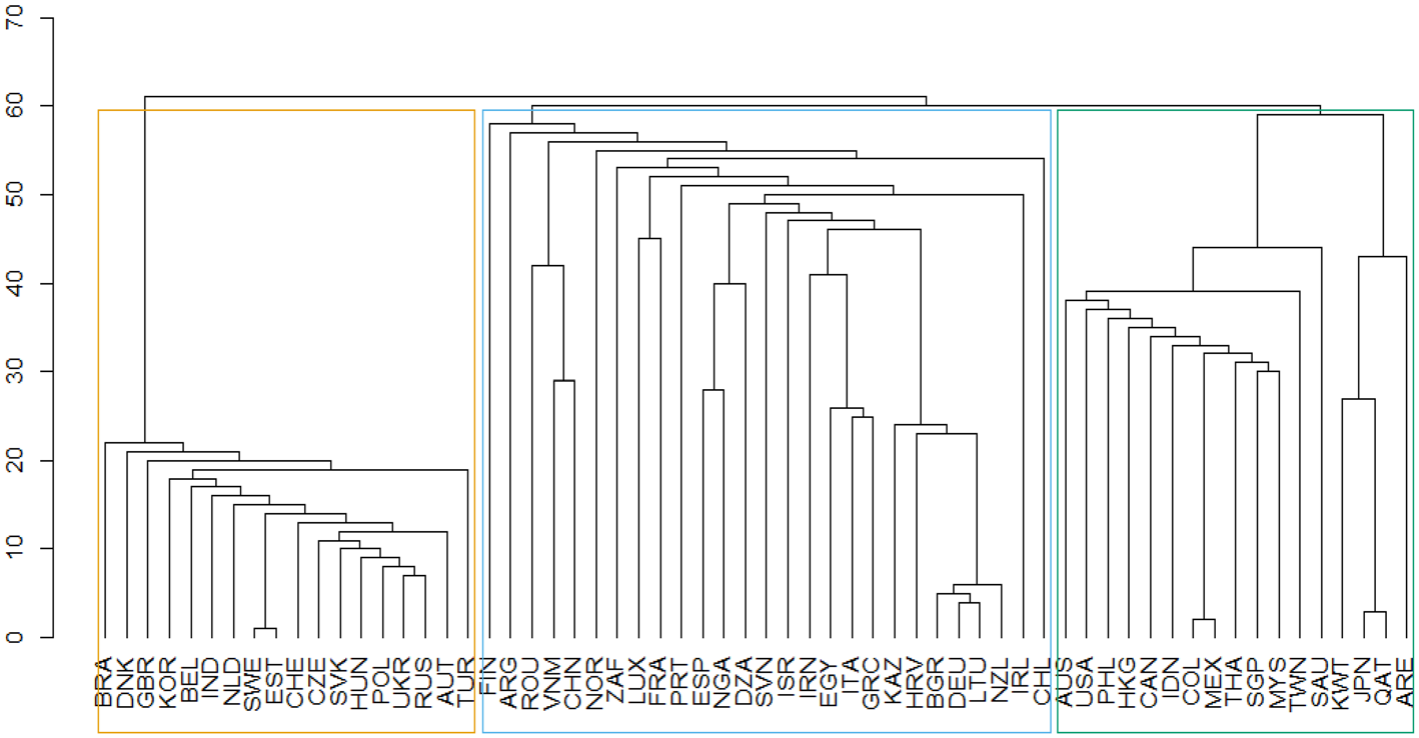
Community detection. The left axis of the dendrogram shows the maximum density links between countries after pairing (Source: Own elaboration with EORA data)

Figure 5 reveals that the network consists of three different communities based on Eq. (16). Community 1 and the less dense includes its majority, East Europe countries, such as Poland, Czech Republic, Belgium, and some predominant nodes as the United Kingdom, Brazil, and Korea, with a total of 18 members. Community 2, the largest one, is led by Spain, France, Germany, Italy, and surprisingly China and includes 27 members. Community 3 includes 17 economies, is denser than community 1, and is led by the United States, Japan, Canada, and Mexico. Due to the integration degree of North America and its relationships with Japan, this last country acts as a bridge connector with countries such as Qatar and then connects to the east Asian countries such as Thailand and Singapore.

As a consequence of community detection, three different aspects arise:

1. The link weights of each community are expected to be correlated with each other due to betweenness centrality, therefore as was reflected in Fig. 3, Germany, which has the top position in the measure, leads the community 2 and is related to France, Italy, and Spain; being the rest of the members the ones connected with these countries. As a consequence, community 2 is mainly at a regional level, predominant with Advanced European countries, but not limited geographically to that territory. In addition, as Azmeh et al. (2022) pointed out, North African countries such as Algeria and Egypt have become important locations for German companies in the reallocation production process as a strategy to seek new low-cost spaces. Therefore, these countries belong to community two, which Germany dominates.
2. Countries with few links inside their communities are more likely to leave the community than those with multiple links. For example, the connection between Mexico and Colombia can be affected if, let us say, Colombia adds a link with Brazil, which belongs to the first community. This evolution happens when a node has a new-stronger weighted link with a country outside of the community. Therefore, the denser the community, the higher the probability they stay together and more probability of having a cluster; otherwise, the community would change and;
3. The use of community detection gives us a broader aspect of how the value-added contribution structures the industry because of its natural production cycle and not the final goods (final consumption which occurs at once), and how even countries that do not belong to the same territory can be in the same community, such as Korea and Brazil.

While GVCs might offer opportunities to upgrade the production process and offers possibilities for new learnings, emerging economies and less developing countries remain in the surrounding area of the network with different types of dependency and regional hubs. Nevertheless, the members of communities are not limited to a geographical area, such as community three, which has America, East Asia, and Oceania countries. Regardless of the results, community detection can help policymakers to conduct specific policies to improve their position in the industry according to members inside and outside the community.

## Concluding remarks

Conducting complex network analysis, this paper applies techniques to visualize and interpret Transport Equipment Industry’s value-added contribution using the Eora Database for 2017.

By quantifying the basic statistical properties of the network, countries are likely to interact with those with similar link structures, the network follows a power-law distribution, which means few, but large countries have dominance in the industry. Therefore, the first conclusion of the research is that the industry is highly centralized and tends to have clusters, which answers the first question addressed in this research.

Subsequently, focusing on visual analysis and applying degree, clustering, and density measures, it was found that, Advanced European countries led the industry, alongside countries like the United States, Japan, and China. The statement is the result of the Euro Zone’s integration in the industry, NAFTA effects, and the continually expanding role of China in the global production system. Other countries with high relevance are Mexico and Brazil in the Americas, Poland and the Czech Republic in East Europe, and Thailand with South Korea in Asia.

Therefore, for the second question of the present study, advanced economies are in their majority the ones in the center, developing countries in which transport equipment is part of the essential industries are located close to the center, while the rest of the countries are far from joining the industry. The network consists of one big cluster with 14 countries and two bridges, one located in East Europe and one in America, suggesting that the governance of the global production from the transport equipment is still regionally even more countries are part of the production chain.

One important aspect of the transport equipment industry is the rapid acceleration of the automatization process. Therefore, the impact of higher automation would create a new network configuration. That is, countries that possess the technology to include it in the production would continue creating a higher value-added in the industry, mostly these countries are the United States, China, and Germany; in contrast developing countries will suffer negatively on the employment side as the automation process focuses on robot adoption instead of the low-educated machine operators. Such is the case of Mexico, in which, according to the study conducted by Artuc et al. (2019), the increasing exposure to the continuous use of robots by the United States industries is affecting the export growth of Mexico to the United States, its principal market.

Centralities measures were used to see the country’s effect on others and the influence of the surrounding countries. In particular, the emergence of European countries was the most influential countries not only because of their position on the network structure, but also for the influence they received from their neighbors.

Nevertheless, the increasing participation of China and the recent tension between the United States and China would reconfigure the production chains. In addition, as Gereffi et al. (2021) pointed out, the industry now tends to be more concentrated in regional production hubs. These bring a more significant challenge for countries such as Mexico, Poland, the Czech Republic, and Thailand, which have increased their participation in the transport equipment industry through the inflows of FDI, but with a lower impact on the technology side for local producers.

For the case of Thailand, even if the country turned in the last years to a more FDI-led policy in the transport equipment industry, its model should include measures to learn foreign knowledge and production skills to start to develop their market instead of being oriented to the re-exports, if the country wants to be part of the influential countries and the ones that have strong connections too.

At first glance, Mexico appears as part of the “important” countries; however, in reality, the country is part of the bridge between the center and the surrounding areas because of its highly dense value-added flows with countries such as the United States, Canada, Japan, and Germany. Therefore, Mexico should implement policies that help the local manufacturers to absorb the technology from the MNCs. It is expected that in the coming years, the country will upgrade the value chains due to the USMCA rule of the local content in the automobile industry for North America, which would help the country continue being competitive against China in the American market.

Hence, betweenness centrality and eigenvector centrality contain relevant information but may depend on how we interpret the results. The measure implies countries’ dominant positions in the network and can be related to access to innovation, R&D, market access, and strong institutional resources, whereas for emerging countries is translated as lower-cost competitiveness, fewer tariffs, and enough labor force.

Finally, community detection provided more than the traditional image of grouping countries by regions. It captures the different aspects of the links between countries, revealing that countries’ value-added contribution flows are not always consistent with their geographical location. Three different communities were detected, led by the United Kingdom, Germany, and the United States.

Nevertheless, even though some countries attract global firms by offering low-cost labor to be integrated into the GVCs, they do not add value to the production process. Suppose they do not support their local firms with policies such as the domestic content on their products or industrial policies that allow local firms to absorb the learning process, such as the success case of China in different industries. In that case, they will remain dependent on the other actors and continue to be part of the assembly-production process.

In the context of the GVCs, the decision of dominant firms to reallocate their production process has a high impact on the value-added content of other countries, whereas it is from the re-exports side or import side. A clear example is Germany; in the last years, they have adopted a more “local” production process to keep the value-added within the country or close areas such as Poland and the Czech Republic due to the lower costs, or United States, Japan, China that has been focusing in the technology with higher investment in R&D.

As discussed throughout the study, analysis based on the GVC using network analysis will allow countries for a much more detailed understanding of their position in the industry as it provides a deeper understanding of the structural form according to other countries’ interactions. Therefore, from this perspective, network science is a valuable tool for adopting proper policies in the trade agenda. Future research can include “cascade falls” phenomenon in the context of the US–China trade war and the COVID-19 pandemic to understand how resilient countries are according to their position in the networks and how much their influence affects others under such circumstances.

## Data Availability

The data and the R studio files that support the findings of this study are available from the corresponding author upon reasonable request.

## Appendix

See Table 3.

## Abbreviations

BACI-CEPII: Base pour l'Analyse du Commerce International-Centre d'Etudes Prospectives d'Informations Internationales
DVA: Domestic value-added
DVX: Indirect value-added
FDI: Foreign Direct Investment
FVA: Foreign value-added
GVC: Global Value Chains
IO: Input–output
MNCs: Multinational companies
MRIOs: Multi-Region Input–Output
NAFTA: North America Free Trade Agreement
RTA: Regional Trade Agreements
R&D: Research and development
